# Naive B Cell Output in HIV-Infected and HIV-Uninfected Children

**DOI:** 10.1089/aid.2018.0170

**Published:** 2019-02-26

**Authors:** Helen Payne, Gabriel Chain, Stuart Adams, Patricia Hunter, Natasha Luckhurst, Kimberly Gilmour, Joanna Lewis, Abdel Babiker, Mark Cotton, Avy Violari, Diana Gibb, Robin Callard, Nigel Klein

**Affiliations:** ^1^ UCL Great Ormond Street Institute of Child Health, London, United Kingdom.; ^2^ Clinical Trials Unit, Medical Research Council, London, United Kingdom.; ^3^ Department of Immunology, Kingston University, London, United Kingdom.; ^4^ CoMPLEX, UCL, London, United Kingdom.; ^5^ Children's Infectious Diseases Clinical Research Unit, Department of Paediatrics and Child Health, Stellenbosch University, Cape Town, South Africa.; ^6^ Perinatal HIV Research Unit, Faculty of Health Sciences, University of the Witwatersrand, Johannesburg, South Africa.

**Keywords:** HIV, ART, child, naive B cell output, bone marrow, KRECs

## Abstract

In this study, we aimed to quantify KREC (kappa-deleting recombination excision circle) levels and naive B cell output in healthy HIV-uninfected children, compared with HIV-infected South African children, before and after starting ART (antiretroviral therapy). Samples were acquired from a Child Wellness Clinic (*n* = 288 HIV-uninfected South African children, 2 weeks–12 years) and the Children with HIV Early Antiretroviral Therapy (CHER) trial (*n* = 153 HIV-infected South African children, 7 weeks–8 years). Naive B cell output was estimated using a mathematical model combining KREC levels to reflect B cell emigration into the circulation, flow cytometry measures of naive unswitched B cells to quantify total body naive B cells, and their rates of proliferation using the intracellular marker Ki67. Naive B cell output increases from birth to 1 year, followed by a decline and plateau into late childhood. HIV-infected children on or off ART had higher naive B cell outputs than their uninfected counterparts (*p* = .01 and *p* = .04). This is the first study to present reference ranges for measurements of KRECs and naive B cell output in healthy and HIV-infected children. Comparison between HIV-uninfected healthy children and HIV-infected children suggests that HIV may increase naive B cell output. Further work is required to fully understand the mechanisms involved and clinical value of measuring naive B cell output in children.

## Introduction

In HIV-infected individuals, CD4 T cell loss results in profound immunodeficiency and susceptibility to infection^1^; however, loss of CD4 T cells can be partially reversed by antiretroviral therapy (ART). In adults this is largely due to peripheral proliferation of an existing T cell repertoire; however, in children the thymus plays a more prominent role in immune reconstitution with the generation of naive T cells.^2^

Naive T cell output is higher in children than adults, increasing in the first year of life and subsequently declining with age^2^; however, a reduction in the output of naive T cells from the thymus in HIV-infected infants and young children has been described.^2^ This is likely to compromise immune reconstitution and contribute to the high rates of mortality seen in HIV-infected children in the first year of life before early ART became standard of care.^3^

While there is increasing understanding of the importance of naive T cell output in immune reconstitution in pediatric HIV, very little is known about the role of naive B cell output in HIV infection and immune recovery. Recognized effects of HIV on B cell immunity include polyclonal hypergammaglobulinemia,^4^ reduction of peripheral memory B cells,^5^ and impaired humoral immunity. Naive B cells play key roles in the immune response to infection, largely through differentiation into antibody producing plasma cells and generation of memory B cells^6^; however, it has not been determined whether naive B cell output changes with HIV infection or even with age in the way that thymic output does.

Quantifying naive B cell output is complex since naive B cell output is a dynamic process. Naive B cells emigrate from the bone marrow and may undergo several different potential fates: proliferation, death, and differentiation. Since B cell development shares some features with T cells, especially in terms of the somatic recombination of the Ig loci, the mathematical models used to quantify thymic outputs^7^ may be applicable for assessing naive B cell output.

Thymic output has previously been assessed by measuring T cell receptor excision circle concentration (TRECs) as a marker of naive T cell production.^8^ TRECs are DNA segments produced during T cell receptor recombination and are stable episomal DNA structures persisting through cell division. However, TRECs will be altered by thymic export, cell division, and longevity of thymic emigrants and cannot give a strict measure of thymic export. Therefore, a mathematical model combining total body naive T cells, rates of naive T cell proliferation, and measurements of TRECs from purified naive CD4^+^ T cells has been developed to provide a more accurate method for determining thymic output.^9^

Since naive B cell output is subject to the same dynamic processes as naive T cell output, we have elected to use a modified thymic output formula to explore naive B cell output. We have used kappa-deleting recombination excision circles (KRECs) instead of TRECs. KRECs are double-stranded DNA structures produced during VJ kappa gene recombination of the precursor B cells' receptor. HIV-infected adults have been shown to have reduced levels of KRECs, which indicate a reduction in naive B cell output^10^; however, no studies have attempted to explore this relationship within a pediatric population.

In this study, we explore how naive B cell output changes throughout childhood and adolescence in two large cohorts of healthy HIV-uninfected and HIV-infected South African children, and how these changes may be affected by HIV infection and ART. Naive B cell output was also calculated from a small group of children from the United Kingdom.

## Materials and Methods

### Participants

Two patient cohorts were used in this study as follows: HIV-uninfected healthy South African children from the “Child Wellness Clinic” (CWC) and HIV-infected children from the “Children with HIV Early Antiretroviral Therapy (CHER) Trial.”^11^ All studies were performed in accordance with good clinical practice and were approved by independent ethics committees: Health Research Ethics Committee of Stellenbosch University (M12/01/005) and Witwatersrand University (040703).

The “Child Wellness Clinic” was established in the informal settlement of Wesbank, Eastern subdistrict of Cape Town, to acquire HIV-uninfected blood specimens to establish hematological and immunological reference ranges in healthy South African children.^12^ Criteria for CWC recruitment required the child to: have no chronic medical conditions, be registered at Wesbank health clinic, and to attend with their biological mother and handheld medical record card. Children aged 2 weeks to 12 years attended once only, and 288 samples were acquired for the measurement of naive B cell output.

The CHER trial randomized HIV-infected infants <12 weeks old with CD4% ≥ 25% to early limited ART for 40 or 96 weeks (ART-40W, ART-96W) or ART deferral until CD4% <25% or clinical disease progression (ART-Def). For all three arms: mean Log_10_ viral load was between 5.6 and 5.7; average baseline weight was 4.4 kg. Ninety percent of these children had mothers who were receiving ART; 81% were within CDC classification *N*; and 20% were breastfed.^1–13^

ART-Def commenced ART at median 27 weeks of age and stayed on continuous ART. ART-96W initiated ART at median 7 weeks continuing ART for 96 weeks then interrupting ART for a median of 70 (IQR 35–109) weeks. Following ART interruption in ART-40W/ART-96W, ART was restarted for clinical progression or CD4% <25%.^11^ Children were reviewed clinically every 4 weeks until week 24, then every 8 weeks until week 48, and every 12 weeks thereafter. Alongside routine clinical blood tests, blood samples were stored for research purposes, 153 of which were used to measure naive B cell output (14 ART-Def and 139 ART-96W). Samples from ART-96W were used from enrolment and the following weeks of the trial (bracketed numbers indicate number of samples at each time point): 4 (34), 12 (16), 40 (25), 60 (16), 96 (20), 248 (18), and 360 (10), equivalent to 7 weeks–8 years of age.

In addition, naive B cell output from a small group of healthy United Kingdom children from Great Ormond Street Hospital was available for comparison with healthy South African children (Supplementary Data, Supplementary Fig. S4; Supplementary Data are available online at www.liebertpub.com/aid).

### Specimen preparation

All blood samples were stabilized with EDTA and isolated as peripheral blood mononuclear cells (PBMCs) using Lymphoprep gradient separation. All CWC blood samples (2–3 mL) were analyzed for HIV-antibody status using the Alere Determine^®^ rapid test (Massachusetts), and full blood cell count was performed by the commercial laboratory (BARC, Johannesburg, South Africa). PBMC aliquots were cryopreserved in 10% dimethyl sulfoxide (DMSO) and 90% fetal calf serum (FCS) at 5–15 × 10^6^ cells/mL (Clinical Laboratory Services^®^). Samples were placed in the Thermo Scientific Nalgene freezing container for 24–48 h at −80°C and then transferred to liquid nitrogen. Specimens were thawed in a water bath at 37°C and gently resuspended in 60% FCS and 40% Roswell Park Memorial Institute (RPMI) medium. Cells were washed twice in RPMI with 2% FCS to remove traces of DMSO, counted and divided for use.

### Flow cytometry and DNA extraction

Immunophenotyping and Ki67 quantification were performed on thawed PBMCs using anti-human fluorochrome-conjugated antibodies (Supplementary Table S1; Flow cytometry Antibodies) and Fixable Viability Dye eFluor^®^660 (Invitrogen, BioLegend, or eBioscience) for 30 min at 4°C before fixation and permeabilization with FOXP3 fixation/permeabilization kit (eBioscience). Intracellular staining with Ki67 was subsequently performed for 30 min at 4°C. Cell analysis was performed on an LSRII or FACSCanto flow cytometer (BD Bioscience) using FACSDiva software (BD Biosciences), and data were analyzed using FlowJo software (Tree Star, Ashland, OR) using Fluorescence Minus One controls. Naive B cells were identified as those expressing CD19, IgD^+^, and CD27^−^, and proliferating naive B cells were identified by expression of the intracellular marker Ki67 (Supplementary Fig. S1).

DNA was extracted from isolated PBMCs using the QIAGEN^®^ QIAamp DNA Extraction Kit (Hilden, Germany) and stored at −20°C. KRECs were quantified from extracted DNA by real-time polymerase chain reaction (PCR) using the Applied Biosystems 7900HT Fast Real-Time PCR System (TaqMan; Life Technologies, Carlsbad, CA), which amplified KRECs and a housekeeping gene (T cell receptor alpha-constant). For each PCR the assay includes 0.5 μL of each primer at a concentration of 45 μM, 0.5 μL fluorogenic probes at a concentration of 10 μM, 6 μL nuclease free water, and 12.5 μL TaqMan Universal PCR Master Mix. PCR conditions were 50°C for 2 min, 95°C for 10 min and then 40 cycles of 95°C for 15 s and 60°C for 1 min (Supplementary Table S2; Primer and probe sequences for KREC PCR).

### Mathematical modeling of naive B cell output

Naive B cell output was determined using flow cytometry measures of total body naive B cells, Ki67 percentage of naive T cells, and quantification of KRECs, applied to the following mathematical formula.^1^

\begin{align*}
{ \rm { \;Naive \;B \ cell \;output \; } } \left( { { \rm { cells \;da } } { { \rm { y } } ^ { { \rm { - 1 } } } } } \right) \theta \left( t \right) = { \frac { y \times N \times \tau }  { \Delta \left( { c - \tau } \right) } } \tag { 1 } 
\end{align*}

Our formula aims to account for proliferation, cell death, and conversion to memory B cells by quantifying the number of total body naive B cells with KREC content at the time of blood sampling, adjusted by percentage of cells that are proliferating. *y* is the fraction of naive B cells expressing Ki67 at time *t*, and $\tau$ (tau) is the total KREC content of PBMCs in the periphery. *N* is the total size of the naive B cell pool, estimated by multiplying the number of naive B cells per microliter of blood by the total volume of blood (μL) [0.97 × Log(body weight in kg) +4.93], then dividing this figure by 0.02 as blood lymphocytes account for ∼2% of the body's total lymphocyte population.

Total body naive B cells with KREC content, adjusted by proliferation, are divided by an estimate of how many divisions have taken place since the naive B cell left the bone marrow. This estimate has been determined by the difference between KREC content as it leaves the bone marrow and KREC content in the periphery at the time at which the blood sample was taken ($c - \tau$), multiplied by the average division rate (Δ = 0.52/day)^1^ of naive B cells. *c* represents KREC content of naive B cells entering the peripheral pool (*c* = 0.6).^1^ The constants *c* and Δ are based on values used for naive T cell output in the absence of published data for naive B cell division time and mean KREC content.

### Statistical analysis

A nonlinear curve was fitted to the naive B cell output data acquired from healthy controls and CHER participants. The nonlinear curve was generated using quantile regression which is robust to unusually-shaped distributions. The confidence intervals shown relate to the median as opposed to enclosing 95% of the data points. Naive B cell output has been compared between groups using Welch *t*-test and Mann–Whitney/Wilcoxon in unadjusted analyses. All analyses were performed using the R software package version 2.15.1^14^ (code available on request).

## Results

### Naive B cell output reference ranges

Age-specific reference ranges from healthy South African children of KRECs and naive B cell output in naive B cells per day are represented in Table 1. The tables show age–specific KREC values represented in four different formats: (1) KRECs per PBMC, (2) KRECs per milliliter of blood, (3) KRECs per million B cells (CD19^+^ lymphocytes), and (4) KRECs per million naive B cells (CD19^+^IgD^+^CD27^−^ lymphocytes). The components of naive B cell output are presented in Supplementary Table S3 and demonstrated in Supplementary Figure S2 suggesting that all three components (total naive B cells, KRECs per PBMC, and Ki67% of naive B cells) may be marginally higher in HIV-infected children on ART in the first 2 years of life compared with healthy HIV-uninfected children.

**Table 1.** Reference Ranges for Kappa-Deleting Recombination Excision Circles and Naive B Cell Output from Healthy HIV-Uninfected and HIV-Infected South African Children

		*KRECs per PBMC*	*KRECs per milliliter of blood*	*KRECs per million B cells*	*KRECs per million naive B cells*	*Naive B cell output (cells/day)*
*Age group*	n	*Median [IQR] (5th–95th centiles)*				
HIV-uninfected healthy children						
0–3 months	33	0.03 [0.02–0.09] (0.01–0.32)	3.5 × 10^5^ [2–7 × 10^5^] (1.2 × 10^5^–1.7 × 10^6^)	2.2 × 10^5^ [1–4 × 10^5^] (5.6 × 10^5^–1 × 10^6^)	2.1 × 10^5^ [1–4 × 10^5^] (6 × 10^4^–7.9 × 10^5^)	3.2 × 10^8^ [1.9–8.3 × 10^8^] (2.2 × 10^7^–1.6 × 10^9^)
3–6 months	33	0.05 [0.02–0.09] (0.005–0.21)	3.9 × 10^5^ [1.7–6.9 × 10^5^] (3.3 × 10^4^–1.7 × 10^6^)	2.1 × 10^5^ [1.1–3.8 × 10^5^] (2.8 × 10^4^–1.1 × 10^6^)	2 × 10^5^ [1.1–3.7 × 10^5^] (1.1 × 10^4^–5.5 × 10^5^)	7.4 × 10^8^ [2 × 10^8^–3.7 × 10^9^] (2 × 10^7^–1 × 10^10^)
6–12 months	50	0.04 [0.02–0.06] (0.002–0.12)	3.4 × 10^5^ [1.7–8.5 × 10^5^] (4 × 10^4^–1.6 × 10^6^)	1.6 × 10^5^ [1.2–3.3 × 10^5^] (1.4 × 10^4^–9.6 × 10^5^)	1.7 × 10^5^ [1–2.3 × 10^5^] (1.2 × 10^4^–4.7 × 10^5^)	6.6 × 10^8^ [1.5 × 10^8^–2.1 × 10^9^] (2.6 × 10^7^–6.8 × 10^10^)
12–24 months	68	0.03 [0.01–0.06] (0.003–0.16)	2.8 × 10^5^ [9.7 × 10^4^–6.4 × 10^5^] (2.4 × 10^4^–1.6 × 10^6^)	1.4 × 10^5^ [7.4 × 10^4^–2.9 × 10^5^] (1 × 10^4^–6.2 × 10^5^)	1.5 × 10^5^ [7.2 × 10^4^–3 × 10^5^] (1.1 × 10^4^–7 × 10^5^)	4 × 10^8^ [1 × 10^8^–2.3 × 10^9^] (2.4 × 10^7^–1 × 10^10^)
2–6 years	80	0.02 [0.009–0.04] (0.002–0.15)	1.1 × 10^5^ [6.1 × 10^4^–4.2 × 10^5^] (1.5 × 10^4^–1.4 × 10^6^)	8.9 × 10^4^ [5.5 × 10^4^–3.1 × 10^5^] (1.2 × 10^4^–1.1 × 10^6^)	1.2 × 10^5^ [7 × 10^4^–2.7 × 10^5^] (1.7 × 10^4^–9.3 × 10^6^)	1.4 × 10^8^ [4.9 × 10^7^–4.3 × 10^8^] (3.8 × 10^6^–4.4 × 10^9^)
6–12 years	24	0.02 [0.008–0.03] (0.001–0.05)	9.7 × 104 [4.1 × 104–1.8 × 105] (9.9 × 103–4.8 × 105)	1.3 × 105 [8.1 × 104–3.4 × 105] (2.6 × 104–5.2 × 105)	1.9 × 105 [9.2 × 104–4.8 × 105] (4.2 × 104–16.6 × 105)	9 × 107 [1.3 × 107–1.7 × 108] (9.4 × 105–6 × 108)
HIV-infected children: ON ART						
0–3 months	34	0.07 [0.04–0.1] (0.01–0.17)	NA	3.8 × 10^5^ [1.8–4.4 × 10^5^] (4.5 × 10^4^–6.8 × 10^5^)	4.3 × 10^5^ [2.0–5.0 × 10^5^] (5.0 × 10^4^–7.3 × 10^5^)	2.2 × 10^9^ [8.4 × 10^8^–4.6 × 10^9^] (2.9 × 10^8^–1.3 × 10^10^)
3–6 months	16	0.07 [0.02–0.09] (0.01–0.21)	NA	2.5 × 10^5^ [1.7–3.8 × 10^5^] (8.4 × 10^4^–7.8 × 10^5^)	2.7 × 10^5^ [1.9–4.2 × 10^5^] (9.4 × 10^4^–9.1 × 10^5^)	2.9 × 10^9^ [2.0 × 10^8^–5.3 × 10^9^] (4.8 × 10^7^–2.5 × 10^10^)
6–12 months	25	0.05 [0.04–0.14] (0.00–0.45)	NA	2.9 × 10^5^ [1.3–5.2 × 10^5^] (2.4 × 10^4^–2.0 × 10^6^)	3.2 × 10^5^ [1.7–5.7 × 10^5^] (2.7 × 10^4^–2.3 × 10^6^)	2.5 × 10^9^ [4.4 × 10^8^–8.0 × 10^9^] (6.3 × 10^7^–6.8 × 10^10^)
12–24 months	16	0.04 [0.02–0.08] (0.01–0.16)	NA	2.0 × 10^5^ [1.3–3.2 × 10^5^] (5.5 × 10^4^–6.0 × 10^5^)	2.5 × 10^5^ [1.6–3.6 × 10^5^] (6.1 × 10^4^–6.9 × 10^5^)	1.0 × 10^9^ [4.0 × 10^8^–4.1 × 10^9^] (6.8 × 10^7^–2.1 × 10^10^)
2 years	20	0.02 [0.01–0.05] (0.01–0.06)	NA	1.1 × 10^5^ [6.1 × 10^4^–1.9 × 10^5^] (5.8 × 10^4^–2.3 × 10^5^)	1.4 × 10^5^ [7.5 × 10^4^–2.5 × 10^5^] (7.1 × 10^4^–3.0 × 10^5^)	5.2 × 10^8^ [1.5 × 10^8^–1.2 × 10^9^] (5.9 × 10^7^–3.2 × 10^9^)
4–5 years	18	0.01 [0.01–0.02] (0.00–0.03)	NA	6.8 × 10^4^ [4.7 × 10^4^–1.2 × 10^5^] (3.9 × 10^4^–1.8 × 10^5^)	9.8 × 10^4^ [6.3 × 10^4^–1.5 × 10^5^] (6.0 × 10^4^–2.7 × 10^5^)	3.6 × 10^7^ [1.5 × 10^7^–1.0 × 10^8^] (1.1 × 10^7^–4.1 × 10^8^)
6–8 years	10	0.02 [0.01–0.02] (0.01–0.06)	NA	9.9 × 10^4^ [8.3 × 10^4^–1.6 × 10^5^] (3.8 × 10^4^–4.4 × 10^5^)	1.6 × 10^5^ [1.3–2.2 × 10^5^] (5.6 × 10^4^–6.0 × 10^5^)	8.6 × 10^7^ [4.4 × 10^7^–3.2 × 10^8^] (5.4 × 10^6^–2.1 × 10^9^)
HIV-infected children: NOT ON ART						
0–3 months ART-Def	15	0.05 [0.03–0.06] (0.02–0.180)	NA	2.4 × 10^5^ [1.5–3.7 × 10^5^] (9.3 × 10^4^–5.9 × 10^5^)	2.8 × 10^5^ [1.7–4.2 × 10^5^] (1.1–6.4 × 10^5^)	7.8 × 10^8^ [3.7 × 10^8^–3.0 × 10^9^] (1.3 × 10^8^–8.6 × 10^9^)

“On ART” means at least 4 weeks on ART. Data are not available for KRECs per milliliter blood as milliliters of blood not recorded on the stored CHER HIV specimens.

ART, antiretroviral therapy; CHER, Children with HIV Early Antiretroviral Therapy; KREC, kappa-deleting recombination excision circle; ON, on ART at least 4 weeks; PBMC, peripheral blood mononuclear cell.

### Naive B cell output with age

**FIG. 1.**
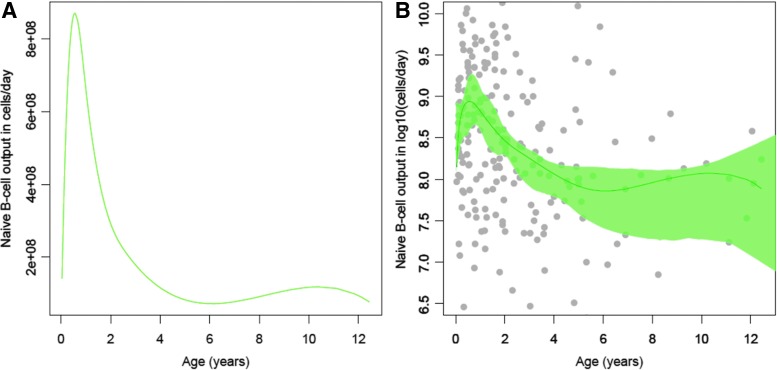
Naive B cell output nonlinear regression curves across age from healthy South African children (CWC, *n* = 288). **(A)** Regression curve representing median naive B cell output in cells per day. **(B)** Logarithmic curve with simultaneous 95% CIs surrounding the centrally *fitted line* and *gray circles* representing each data point. CIs, confidence intervals; CWC, Child Wellness Clinic.

In healthy children, naive B cell output changes with age, exhibiting a steep increase and peak in the first year of life (median 8 × 10^8^ naive B cells/day), followed by a rapid decline by 4 years of age (median 7.2 × 10^7^ naive B cells/day) and plateau at 12 years (Figs. 1A and 1B).

### Naive B cell output in HIV-uninfected versus HIV-infected children

**FIG. 2.**
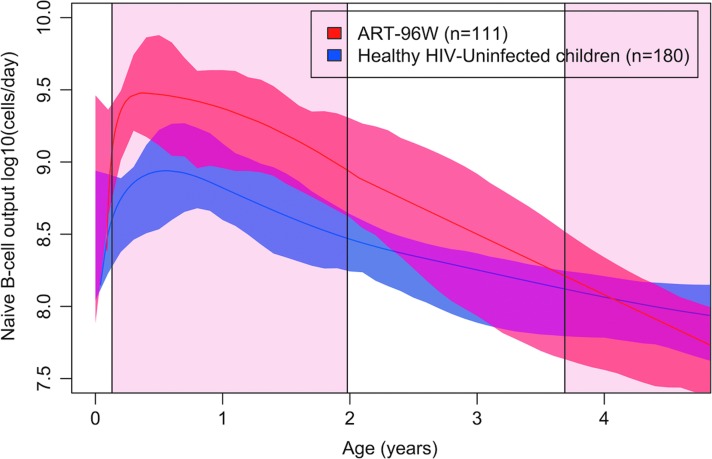
Age-specific naive B cell output (median) nonlinear regression curves of HIV-uninfected children (CWC, *blue*, *n* = 180) compared to HIV-infected children (ART-96W, *red*, *n* = 111 samples in 70 individuals) until 5 years of age. ART-96W initiated ART at median 7 weeks until 2 years, interrupting ART for a median of 89 (IQR 17–145) weeks in these 70 children. The *central line* is the best fit of the data, and surrounding *shaded* areas are 95% CIs of the regression curve. The *pink* shaded area indicates planned ART treatment, *white* areas indicate planned breaks in treatment. ART, anti-retroviral therapy.

Measurements of naive B cell output from children treated with ART for 96 weeks (ART-96W) were compared to healthy HIV-uninfected South African children from the CWC. Figure 2 illustrates that ART-treated HIV-infected children (*n* = 111) had higher naive B cell output than their HIV-uninfected counterparts (*n* = 180) overall (Welch's *t*-test *p* < .0001), particularly up to the age of 2 when they were on ART (*p* = .01).

In the naive B cell output curve for healthy children, every sample used from the CWC was from a different child; however, in ART-96W 111 samples were used from 70 children. To assess whether the presence of duplicates affected the shape and magnitude of the naive B cell output curve duplicates were removed at random and the curve was replotted, demonstrating minimal change in the shape and quantity of naive B cell output overall for the line of best fit and surrounding 95% confidence intervals (Supplementary Fig. S3).

### Naive B cell output in deferred-ART versus HIV-uninfected children aged 0–3 months

**FIG. 3.**
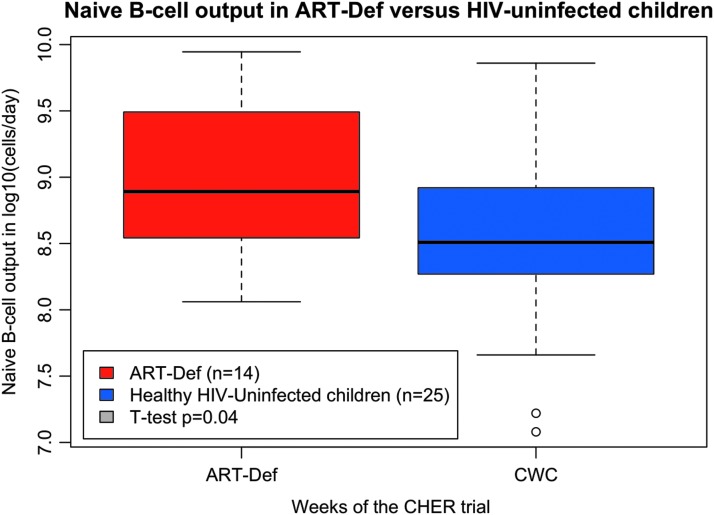
The effect of deferred ART on naive B cell output between 0 and 3 months of age compared with healthy HIV-uninfected children (ART-Def *n* = 14, *red*; CWC *n* = 25, *blue*; *t*-test *p* = .04).

Children who deferred ART as per The CHER Trial randomization, aged 0–3 months, had significantly higher naive B cell output compared to healthy HIV-uninfected children of the same age (Fig. 3, ART-Def *n* = 14, CWC *n* = 25, *p* = .04).

## Discussion

This is the first study to present a reference range of KRECs in a large group of healthy children. This could be a valuable resource within clinical environments that might utilize the measurement of KRECs for primary immunodeficiency diagnostics, recovery from bone marrow transplant, surveillance for leukemia relapse, and potential assessment of immunological health in HIV-infected children.

In this study, we have presented four different representations of the KREC data: KRECs per PBMC, KRECs per milliliter blood, KRECs per million B cells, and KRECs per million naive B cells, and these measures have value in some clinical settings when it may not be practical to quantify Ki67. Previous studies estimating bone marrow output have used these measures; however, these measures do not account for KREC dilution upon cell division.^15,16^ Our adapted equation estimates naive B cell output more accurately than KREC values alone as it accounts for both B cell proliferation and KREC content. Taking into consideration the rate of naive B cell proliferation in the periphery may be a more biologically relevant and more clinically useful measurement in disease states, when cell proliferation is likely to be high such as in HIV infection and other inflammatory states.

Two assumptions were made in the adapted equation that need further qualification for naive B cells. The first is the time taken for a single naive B cell to proliferate. Studies have calculated the average times of B and T cell division as between 8 and 60 h^17,18^; however, a concrete figures does not exist for this parameter as division times can change depending on levels of immune activation. We decided upon 0.52 divisions per day, based upon T cell values as the most accurate. Second, an average KREC content of prenaive B cells entering the peripheral pool is assumed as 0.6, based on TREC content of immature thymocytes. These two parameters need to be measured to improve accuracy of the model, and further work is required to explore the validity of these values. Although *c* and Δ are key parameters of our calculations, they are constants within the formula and were different values known, they would not affect the differences observed in naive B cell output when comparing the cohorts.

Our study also used a novel method for determining naive B cell output in healthy and HIV-infected children. Exploring the difference between HIV-uninfected children and HIV-infected children serves to try to put this measure in a clinical context and demonstrate the value of accounting for proliferation. In healthy South African children, naive B cell output increased steeply from birth to peak around 1 year of age, followed by a rapid decline until 6 years and minimal further change thereafter. The high naive B cell output coincides with the rapid development of a child's immune system during the first year of life,^19^ and subsequent rapid decline in naive B cell output may reflect a change in the physiological activity of the bone marrow and/or may be influenced by increased trafficking of naive B cells to the lymphoid organs.^20^

In HIV-infected children on or off ART, naive B cell output was significantly higher than their uninfected counterparts in the first 2 years of life. We hypothesize that the differences observed may be due to HIV-induced stimulation of humoral immunity with greater differentiation of naive to memory B cells and higher rates of cell turnover, leading to a compensatory increase in generation of naive B cells from the bone marrow. This is reflected by examining the components of the naive B cell output formula (Supplementary Fig. 2); however, there should be limited interpretation of these components in isolation since they are intrinsically related. Increased proliferation is well recognized in HIV infection, and so assessment of total naive B cells may reflect clonal proliferation and not necessarily naive B cell output. Equally KREC measures in isolation can be misleading since increased rates of proliferation may effectively dilute their concentration. Previous studies have shown that in HIV-infected patients, higher viral load and antigen burden led to higher rates of naive T cell turnover, production, and differentiation into memory and effector cells,^21^ and it may be plausible that a similar mechanism exists for naive B cell output.

One of the limitations of our study was the very low number of samples available from ART-Def during the period while they were off ART. Although no difference in naive B cell output was identified between ART-Deferred compared to early ART, the analysis may have been influenced by the CHER trial study design, which determined that children who deteriorated clinically or immunologically were restarted on ART. Consequently, the design has preselected the children who most likely had better immune systems since they managed to remain off ART for longer.

In summary, we present the first large dataset of KREC measurements and naive B cell output in healthy HIV-uninfected and HIV-infected children from South Africa. The comparison between HIV-infected children from the CHER trial and the HIV-uninfected children suggests that HIV may increase naive B cell output and indicate that a potential feedback mechanism might exists to drive bone marrow output. However, how changes in naive B cell output relate to B cell function and ultimately clinical health remain unknown. Further work is needed to understand how best to preserve naive B cell output in children infected with HIV and in conditions in which B cell dynamics could be impaired such as following treatment for malignancy and congenital immunodeficiency.

## Supplementary Material

Supplemental data
