# Supplementary material for: Naive B Cell Output in HIV-Infected and HIV-Uninfected Children
Source: AIDS Res Hum Retroviruses. 2019 Feb 26;35(1):33–9. doi: 10.1089/aid.2018.0170 (PMC6863188; doi:10.1089/aid.2018.0170)
Supplement: Supplemental data [file Supp_Data.zip › Supp_Data.pdf]

Supplementary Data

A summary of the preliminary data from experiments measuring naive B cell output from children from the United Kingdom.

Objectives

In this experiment, we aimed to quantify naive B cell output from children in the United Kingdom and compare them to age-matched healthy African children from the CWC (Child Wellness Clinic).

Materials and Methods

Sixteen samples from United Kingdom children were collected from GOSH (Great Ormond Street Hospital) and used for analysis of naive B cell output. These samples were age matched to 21 samples from the CWC cohort (6–18 years). United Kingdom samples were processed and immunophenotyped on the same day. Cryopreservation was performed with United Kingdom samples; however, in our group we have performed experiments that demonstrate that there is no significant difference between measuring any of these B cell markers, particularly Ki67 (publication in progress). Immunophenotyping, naive B cell output quantification, and statistical analysis were performed as per CWC samples.

Results

Samples from healthy children in the United Kingdom ($n = 16$) were compared to healthy samples from the CWC study in South Africa ($n = 21$) to determine possible geographical differences in naive B cell output. Naive B cell output was significantly higher (Mann–Whitney *t*-test, $p = 0.013$) in children from Africa (median, 1.01×10^8 cells/day) compared to children from the United Kingdom (median, 8.54×10^6 cells/day). It is worth noting that the mean age ranges differ between both cohorts, with the United Kingdom mean age of 14 years (range 6–18) and African mean age of 9 years (range 6–12). Within each cohort, there is little change in naive B cell output with increasing age (figure below).

Supplementary Table S1. List of Antibodies Used in Flow Cytometry Panels

Antibody	*Company*	*Conjugate*
IgD	BD Biosciences	BV510
CD19	BD Biosciences	PerCP-Cy5.5
Ki67	BD Biosciences	PE
CD27	BD Biosciences	BV421
CD3	BD Biosciences	FITC
ef660 Fixable Viability Dye	eBioscience	APC

Discussion

We decided to include these data as supplementary information since we cannot exclude the possibility that our observed changes are due to the differences in the age ranges, sample size, and methodology; however, in this limited analysis British children had significantly lower naive B cell outputs than South African children between 6 and 18 years of age. Previous studies have shown that in HIV-infected patients, higher viral load and antigen burden led to higher rates of naive T cell turnover, production, and differentiation into memory and effector cells.[1] In this study, we hypothesize that, given increased antigen burden in South Africa,[2] there may be greater differentiation of naive to memory B cells and higher rates of cell turnover, and this might lead to a compensatory increase in generation of naive B cells from the bone marrow. As children under the age of 6 years old were not recruited in the United Kingdom, we cannot comment on the dynamics of B cell output in younger United Kingdom children; however, the overall trend in each cohort showed little change in outputs from age 6 to 18 years.

Naive B cell output in healthy United Kingdom and African children. (A) Scatterplot of naive B cell output from samples taken in the United Kingdom ($n = 16$) (*red*) and Africa ($n = 21$) (*black*) in log10 (cells/day). Individual samples are represented by dots and displayed with mean ± standard deviation. (B) Naive B cell output from samples taken in the United Kingdom ($n = 16$) (*red*) and Africa ($n = 21$) (*black*) in log10 (cells/day). United Kingdom $Y = 0.01986*X + 6.788$; $R^2 = 0.009514$. African $y = 0.002207 + 7.682$; $R^2 = 0.0000238$.

Supplementary References

1. Sottini A, Serana F, Bertoli D, *et al.*: Simultaneous quantification of T-cell receptor excision circles (TRECs) and K-deleting recombination excision circles (KRECs) by real-time PCR. J Vis Exp 2014;94:1–10.
2. Mensen A, Ochs C, Stroux A, *et al.*: Utilization of TREC and KREC quantification for the monitoring of early T- and B-cell neogenesis in adult patients after allogeneic hematopoietic stem cell transplantation. J Transl Med 2013;11:1.

Supplementary Table S2. Primers and Probes for the Quantitative Kappa-Deleting Recombination Excision Circle Polymerase Chain Reaction Assay

Primer/probe	*Sequence*
KREC forward	5í-TCC CTT AGT GGC ATT ATT TGT ATC ACT-3í
KREC reverse	5í-AGG AGC CAG CTC TTA CCC TAG AGT-3í
KREC probe	5í-VIC-TCT GCA CGG GCA GCA GGT TGG-TAMRA-3í
TRAC forward	5í-TGG CCT AAC CCT GAT CCT CTT-3í
TRAC reverse	5í-GGA TTT AGA GTC TCT CAG CTG GTA CAC-3í
TRAC probe	5í-FAM-TCC CAC AGA TAT CCA GAA CCC TGA CCC-TAMRA-3í

TRAC, T-cell receptor alpha-constant gene.

Supplementary Table S3. Raw Data of the Components of the Naive B Cell Output Equation

		KRECs per PBMC	*Naive B cells per μL blood*	*Naive B cell Ki67%*
Age group	n		*Median [IQR] (5th–95th centiles)*	
HIV-uninfected healthy children				
0–3 months	33	0.03 [0.02–0.09] (0.01–0.32)	855.00 [665.54–1227.21] (215.03–1962.12)	9.18 [5.90–12.65] (4.50–15.66)
3–6 months	33	0.05 [0.02–0.09] (0.005–0.21)	1358 [990.74–2045.73] (520.67–2850.16)	4.54 [990.74–5.70] (1.50–7.75)
6–12 months	50	0.04 [0.02–0.06] (0.002–0.12)	1063 [662.54–1427.28] (420.96–2547.58)	4 [2.86–5.83] (2.20–10.11)
12–24 months	68	0.03 [0.01–0.06] (0.003–0.16)	939.00 [583.20–1408.71] (370.80–2353.96)	3.48 [2.46–4.37] (1.65–7.23)
2–6 years	80	0.02 [0.009–0.04] (0.002–0.15)	412.00 [239.62–662.38] (81.97–1252.81)	4.26 [3.05–5.87] (1.91–11.10)
6–12 years	24	0.02 [0.008–0.03] (0.001–0.05)	182.00 [104.62–276.01] (48.10–477.53)	4.35 [2.42–5.88] (1.26–8.03)
HIV-infected children: ON ART				
0–3 months	34	0.06 [0.04–0.10] (0.02–0.17)	1682.00 [1348.79–2187.00] (681.07–4272.09)	9.00 [6.34–12.5] (2.92–25.24)
3–6 months	16	0.07 [0.02–0.09] (0.01–0.21)	1682.00 [827.74–2335.59] (676.04–3203.06)	5.00 [4.22–6.37] (3.50–7.42)
6–12 months	25	0.05 [0.03–0.12] (0.01–0.34)	1267.00 [922.81–1705.07] (443.70–2730.42)	4.00 [2.64–4.08] (1.94–5.67)
12–24 months	16	0.04 [0.02–0.08] (0.01–0.16)	997.00 [639.61–1395.04] (351.34–2093.90)	3.00 [2.32–4.33] (1.33–6.73)
2 years	20	0.02 [0.01–0.04] (0.01–0.08)	912.10 [538.29–1053.05] (351.15–1324.80)	2.97 [2.36–3.50] (1.31–3.99)
4–5 years	18	0.01 [0.01–0.02] (0.00–0.03)	273.56 [197.90–396.91] (123.14–878.21)	4.07 [2.76–396.91] (123.14–878.21)
6–8 years	10	0.01 [0.01–0.02] (0.00–0.06)	289.00 [144.93–450.05] (83.07–717.41)	3.00 [1.79–3.39] (0.88–4.99)
HIV-infected children: NOT ON ART				
0–3 months ART-Def	14	0.06 [0.03–0.09] (0.02–0.19)	1638.37 [1006.08–1982.68] (658.88–2476.17)	8.63 [7.42–10.55] (6.04–13.83)

ART, antiretroviral therapy; ON, on ART at least 4 weeks; PBMC, peripheral blood mononuclear cell.
