# Supplementary figures and images for: Naive B Cell Output in HIV-Infected and HIV-Uninfected Children
Source: AIDS Res Hum Retroviruses. 2019 Feb 26;35(1):33–9. doi: 10.1089/aid.2018.0170 (PMC6863188; doi:10.1089/aid.2018.0170)

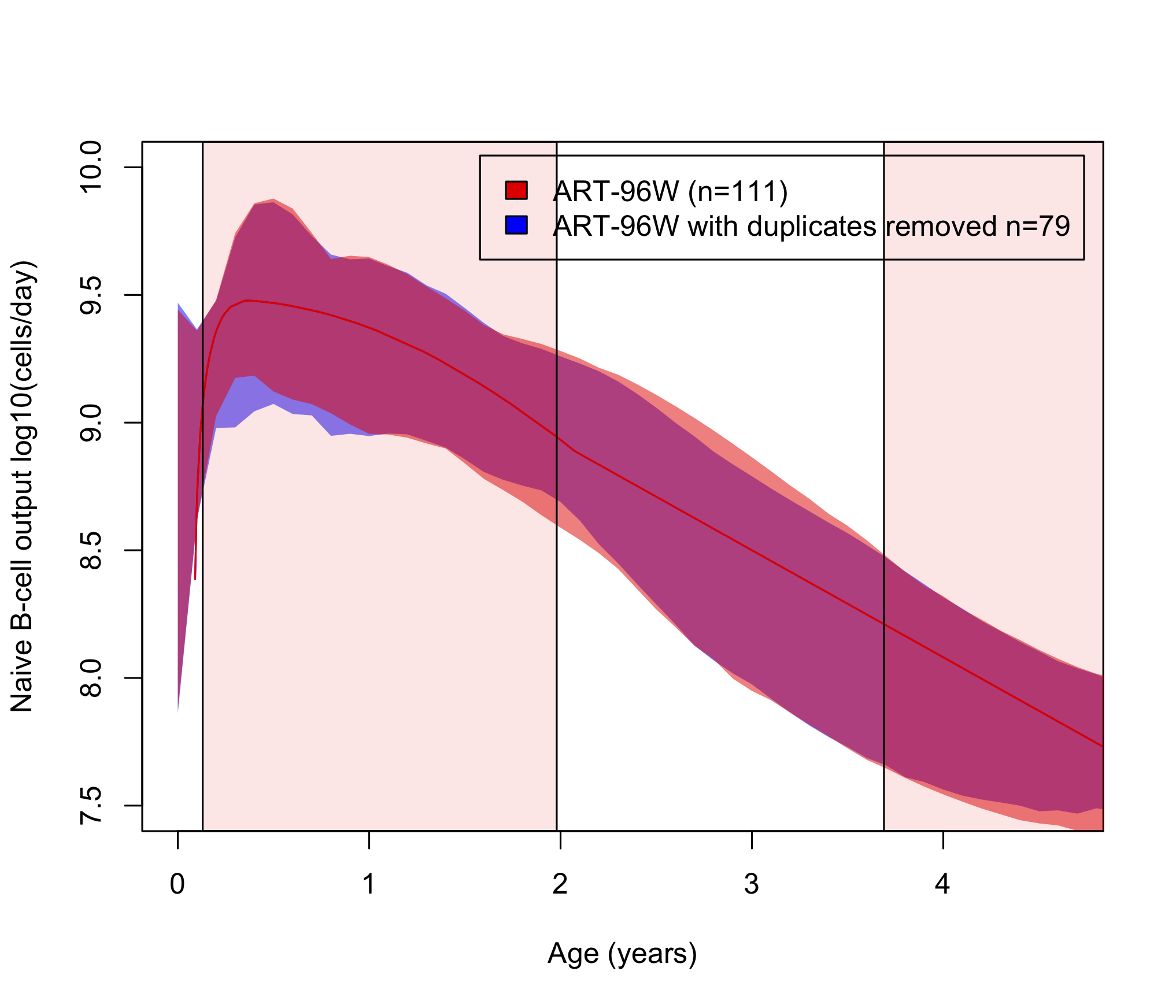

Supplement: Supplemental data [file Supp_Data.zip › Supp_Fig3.tiff]

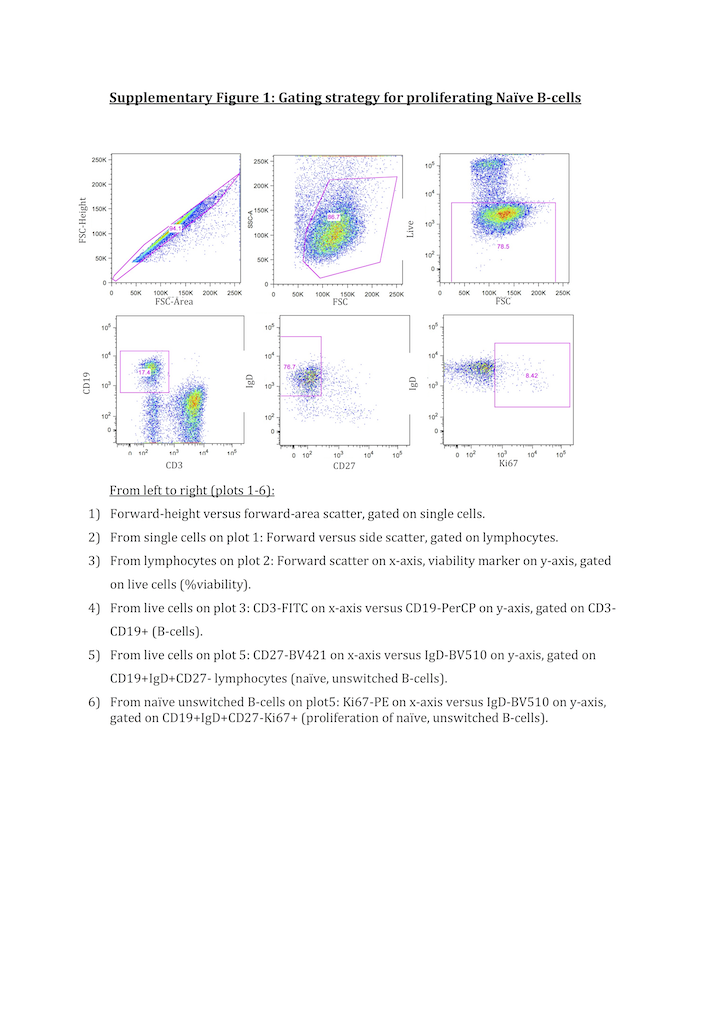

Supplement: Supplemental data [file Supp_Data.zip › Supp_Fig1.tiff]

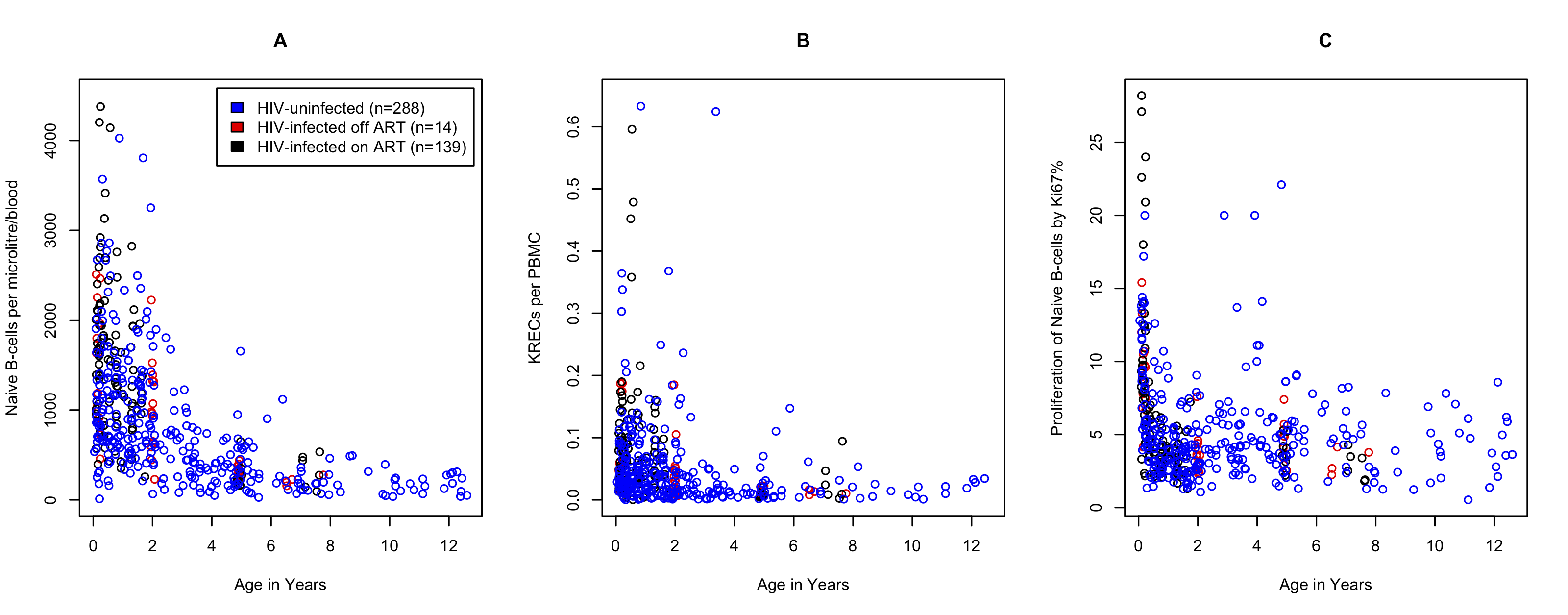

Supplement: Supplemental data [file Supp_Data.zip › Supp_Fig2.tiff]
